# Equity-based carbon neutral plan induces cross-regional coal leakage and industrial relocation

**DOI:** 10.1016/j.isci.2024.109079

**Published:** 2024-02-01

**Authors:** Ziqiao Zhou, Xiaotian Ma, Silu Zhang, Chaoyi Guo, Xiaorui Liu, Lin Zhang, Yang Xie

**Affiliations:** 1 College of Environmental Sciences and Engineering, Peking University, Beijing 100871, China; 2 China Electric Power Research Institute, Beijing 100192, China; 3 School of Energy and Environment, City University of Hong Kong, Kowloon Tong, Hong Kong SAR; 4 Center for Ocean Research in Hong Kong and Macau, Hong Kong, Hong Kong SAR; 5 School of Economics and Management, Beihang University, Beijing 100191, China; 6 Laboratory for Low-carbon Intelligent Governance, Beihang University, Beijing 100191, China

**Keywords:** Earth sciences, Engineering, Energy management, Energy Modeling

## Abstract

China as a major coal-consuming economy faces the challenge of balancing economic development and carbon neutrality goal. This paper incorporates both efficiency-based and equity-based carbon neutrality policies into a numerical model to quantitatively assess how coal reduction under various carbon-neutral policies affects energy mix, economic growth, and industrial structures by 2060. Results show the nationwide coal intensity will ultimately plunge by over 95% from 2017 to 2060, mainly attributed to the coal-phasing-out in most industries. National Gross Domestic Product losses reaches 4,951 billion USD in efficiency-based scenarios by 2060, and the economic losses are even more severe in less developed provinces, especially provinces in Northern China. Although the equity-based policy can reduce the economic burden for the Northern China, the equity-based policy is accompanied by a significant regional shift in coal across the country: eastern coal-intense industries will be relocated northward, leading to increases in embodied coal consumption.

## Highlights

• Six scenarios are designed to fulfill the carbon neutrality target in China
• Nationwide coal intensity drops driven by phased coal reduction across industries
• Efficiency-based scenarios result in GDP loss, which is more sever in the North
• Equity-based policies trigger a shift in coal-related industries toward the North

## Graphical abstract

**Figure:**
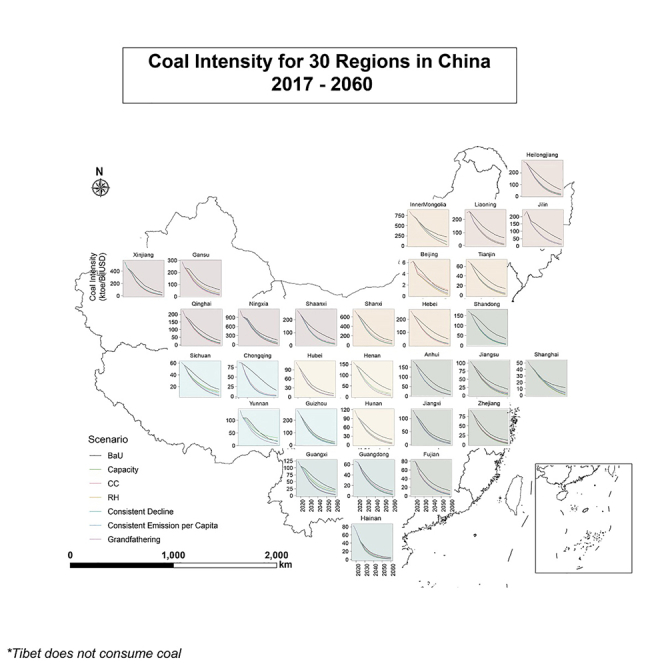

## Introduction

Global warming is threatening our society, calling for worldwide endeavors to reduce greenhouse gas (GHG) emissions. Reducing the consumption of fossil fuels and developing renewable energy are of vital importance to limit the emissions of carbon dioxide (CO_2_). Furthermore, the current carbon pathway still puts humanity on track for more than doubling the 1.5°-Celsius limit admitted in the Paris Agreement,^1^ requiring governments to take action now to secure the well-below 2° temperature target. The requirement is even more urgent for large energy consumers, such as China.

As China’s carbon emissions contribute up to 30% of global emissions, its climate actions can significantly help the world achieve the net-zero target.^2^^,^^3^^,^^4^ However, China’s growing demand in energy imposes new challenges to its energy supply system for reducing carbon. Coal remains the dominant fuel in the Chinese energy structure, even in well-developed cities like Shanghai.^5^^,^^6^^,^^7^^,^^8^^,^^9^ Nevertheless, in 2021, the Glasgow Climate Pact, a global agreement, explicitly included reducing fossil fuel use for the first time. The decision further emphasizes that coal should be gradually phased down.^10^ Hence, balancing economic development and GHG mitigation become a significant challenge for China, especially when coal-fired power is usually the cheapest energy until 2050 without policy intervention.^11^^,^^12^ China’s strong commitment to achieve the neutrality goal primarily focus on quantity-based policies, which are the carbon intensity constraints requiring provinces to lower their carbon emissions per Gross Domestic Product (GDP). To formulate efficient mitigation strategies, policymakers require information on the socioeconomic impacts that potential policy interventions may have on local development. Furthermore, as net-zero emissions does not mean absolute zero emission,^13^ the specific reduction target for coal remains a puzzling issue for policymakers, as it involves the retention and utilization of a portion of coal as alternative options or emergency energy, rather than a complete phase-out.

Phasing down coal is not only a step toward energy transition, but also a key to carbon neutrality in a carbon-constrained economic restructuring process in China. However, prior studies mainly focus on evaluation of coal-related policies. Chinese government used to enact a series of command-and-control policies such as quantity-control and working-day limitations on the coal mining industry to solve overcapacity. However, it is found that these policies either have negative environmental impacts^14^ or lead to a more severe overcapacity of coal resources and skyrocketing coal prices.^15^ One explanation is that China’s unprecedented economic growth rate and increasing demand for energy services imply that its energy consumption is more demand-driven, consistent with past global energy transition experiences. However, demand-driven market mechanisms are not efficient in coal reduction either. The Chinese government launched two market mechanisms for energy industries in pilot provinces, which are energy trading^16^^,^^17^ or coal trading,^18^^,^^19^ and taxation.^20^^,^^21^^,^^22^ Researches show that, first, market mechanisms may encounter some difficulties in practice because search-match costs are too high for both buyers and sellers in China, and the quota unit is not standardized.^18^ A simulated taxation scenario shows that taxation can be effective only in capacity control, but less useful in the promotion of cleaner energy.^20^^,^^23^ Second, current research fails to set a meaningful quantitative target for coal reduction, however, such reduction target is important for policymakers to measure the economic cost and time for the energy transition. The typical way to define a target is based on a percentage of coal in the energy mix, but this varies across research for different purposes.^15^^,^^24^^,^^25^^,^^26^^,^^27^

There is a strong need to evaluate the effectiveness of carbon policies on coal reduction and their socioeconomic impacts on China, especially on coal-reliant provinces and sectors. Therefore, this paper seeks to answer the following questions: (1) To what extent can different types of carbon neutrality policies drive away coal in China? (2) What are the socioeconomic consequences of carbon neutrality induced coal reduction? (3) Can policy interventions alleviate the uneven losses on coal-reliant provinces, and through which channels? To address the previous questions, we propose an integrated assessment framework consisting of a dynamic 31-region computable general equilibrium (CGE) model coupled with environmentally extended input-output analysis (EIOA) and structural path decomposition (SPD). The CGE model, featured in representing the energy- and carbon-intensive sectors of China’s provincial economies, is used to quantitatively assess coal reliance measured by coal consumption per GDP^28^ or sectoral output from 2017 to 2060 under different carbon constraints. We further distinguish the embodied coal consumption in domestic bilateral provincial trade and identify the key drivers of coal reduction through the EIOA and SPD.

Our research unfolds the effectiveness of coal reduction at the provincial and sectoral levels in a carbon-constrained future and identifies the key regions and industries behind such reduction under different scenarios. As other developing countries also have similar overall energy structure, our results offer supports for the socioeconomic feasibility of coal reduction planning in the context of carbon neutrality, not only for China but also for other these countries. We construct six carbon neutrality policy scenarios to evaluate coal decoupling by 2060, based on the principle of efficiency or equity. Results show that in order to achieve the global temperature goal, the coal intensity should decline immediately. With policy intervention, national coal intensity reaches around 7 ktoe/billion USD in 2060 with provincial disparity. However, the national GDP losses exceed 4,951 billion USD in 2060, and Northern China suffers the greatest economic losses. Although equity-based policies can alleviate its losses, the coal intensity and embodied coal consumption in Northern China tend to increase through provincial bilateral trade.

## Results

### Regional and provincial coal reduction: Greater efforts needed in the North

**Figure 1:**
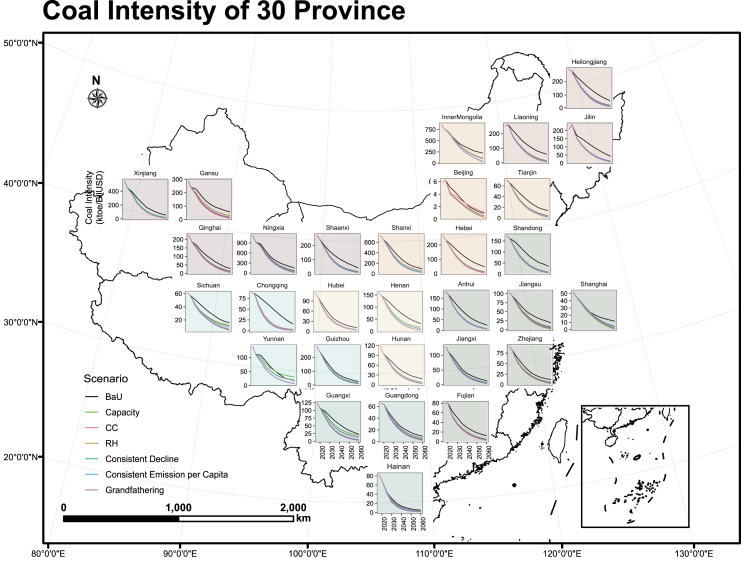
Regional coal intensity 2017–2060.

Coal intensity measures the reliance on coal for economic development. To identify the provinces and industrial sectors that are most reliant on coal, we employed a statistical technique known as the natural break method to effectively reveal distinct categories or clusters within the data. In 2017, China’s coal intensity, measured as thousand tons of oil equivalent per billion U.S. dollars (ktoe/BilUSD), stood at 164 for the nation as a whole. When consider regional disparities, coal intensity across the 30 mainland provinces (excluding Tibet, which consumes no coal in its energy mix) ranges from 6 ktoe/BilUSD in Beijing to 1127 ktoe/BilUSD in Ningxia province. Figure 1 shows the regional coal intensities in 2017 and their modeled trends through 2060, where the red color means the GDP of the province relies more on coal consumption. In order to better describe the geographic pattern of coal intensity, we further divide the 31 provinces into seven geographic regions following the Constitution of China: Northwest (NW), North (N), Northeast (NE), Center(C), Southwest (SW), South (S), and East (E). The precise regional division can be found in appendix Table S2. Generally, coal intensity is more significant in three northern regions (NW, N, and NE). The top five provinces that are most coal-reliant for economic development in 2017 all located in northern regions. Among the highly coal-reliant five provinces, Ningxia, Inner Mongolia, and Shanxi have coal intensity far beyond that of Xinjiang and Gansu.

The coal intensity should start to decrease immediately and drastically, but northern regions rely more on coal for economic development than that of the other regions. In 2060, the nationwide coal intensity will ultimately plunge, and the provincial coal intensities tend to converge. Under the business as usual (BaU) scenario, the national coal intensity of China decreases to 26 ktoe/BilUSD, a decline of approximately 84% from the 2017 level. Without policy interventions, the geographic pattern of coal intensity remains similar to that in 2017, where top ten provinces with largest coal intensity all located in three northern regions. Implementation of carbon neutrality further reduce coal intensity for all provinces by over 95% from 2017 to 2060, and different carbon budget allocation method may alter the geographic pattern of coal intensity. Six policy scenarios are set based on either principle of efficiency or principle of equity. Among six policy scenarios, national coal intensity in 2060 ranges from 7 ktoe/BilUSD under efficiency-based consistent command (CC) scenario to 8 ktoe/BilUSD under equity-based grandfathering scenario. Generally, three northern regions are still required to put greater effort into coal decoupling than other regions, especially under efficiency-based scenarios. However, under equity-based scenarios, which allocate emission budget based on either ability-to-pay method or grandfathering principle, coal intensities of some provinces located in southwest region increase.

Coal intensities of Beijing and Yunnan show unusual response to policy interventions. In the CC scenario, a typical efficiency-based scenario, Beijing’s coal intensity is unexpectedly higher to the baseline in 2060, primarily due to its exceptionally low 2017 coal intensity of 6 ktoe/BilUSD. Our model simulations suggest that Beijing can significantly reduce its coal intensity without additional policy interventions, potentially reaching less than 1 ktoe/BilUSD by 2060. If the carbon neutrality goal requires all provinces to meet the same criteria, there is no doubt that relatively well-developed provinces like Beijing require less effort, and in some extreme cases where other non-coal fuel prices would increase too much, they can even rebound their coal consumption without violating the policy and carbon neutrality goal. To address this issue, equity-based scenarios allocate more carbon budget to less-developed provinces while restricting developed regions like Beijing. However, the coal intensities of Yunnan under consistent emission per capita, capacity and Robin Hood (RH) scenario exceed those under the BaU. A more relaxed carbon constraint enables less-developed regions like Yunnan to develop carbon-intensive industries within their allocated carbon budget.

**Figure 2:**
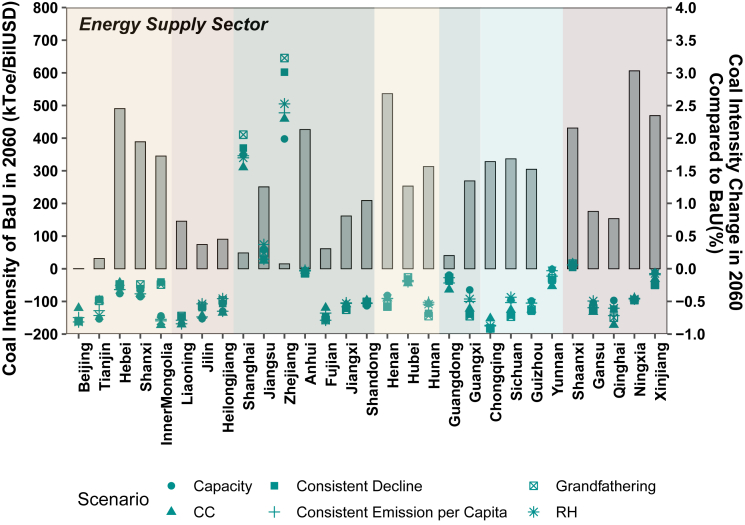
Coal intensity of energy supply sector in 2060 (in which bars stand for the BaU scenario, while points denote different policy scenarios).

In 2060, a noticeable reduction in coal intensity is observed across most industrial sectors under both the BaU and policy scenarios (Figure S1). Some provincial sectors may even become coal-free with policy interventions. However, it is worth highlighting that the energy supply sector stands out as an exception, with certain provinces experiencing a rebound in coal intensity by 2060. Figure 2 focuses on the coal intensity of energy supply sector in 2060 and its comparative reduction in various policy scenarios. In the BaU scenario, the national coal intensity of energy supply sector has a modest decrease from 262 ktoe/BilUSD in 2017 to 249 ktoe/BilUSD by 2060. Notably, there are ten provinces exhibit a rebound in coal intensity within the energy supply sector. Carbon neutrality policies prove effective in curbing this trend in most provinces, others, such as Shanghai (E), Jiangsu (E), Zhejiang (E), Shaanxi (NW), and Xinjiang (NW), still witness an increase in sectoral coal intensity by 2060, even with policy intervention. The root cause of this increase in sectoral coal intensities lies in the fact that the reduction in sectoral output lags behind that of coal consumption. For these provinces, achieving complete decoupling of coal consumption from output in the energy supply sector may be overly challenging. As a result, a gradual reduction in output, alongside reduced coal consumption, becomes the response to the required carbon intensity.

### Uneven GDP losses across provinces under carbon-neutrality policies

Carbon emission constraints have a profound impact on the use of fuel energy, including coal. This, in turn, affects production as well as commodity and energy prices that ultimately influence various socio-economic factors, including GDP, its composition and industrial structure. Therefore, this section primarily focuses on discussing and presenting the socio-economic outcomes resulting from emission constraints and coal reduction.

Generally, it is worth noting that the implementation of equity-based policies tends to result in higher national economic losses compared to their efficiency-based counterparts. Within efficiency scenarios, the average national GDP loss in 2060 amounts to 4,951 billion US dollars (BilUSD), whereas equity-based scenarios yield an average loss of 5,643 BilUSD. At the level of GDP per capita, the value decreases by 8.20% and 9.69% in the CC and RH scenarios, respectively.

**Figure 3:**
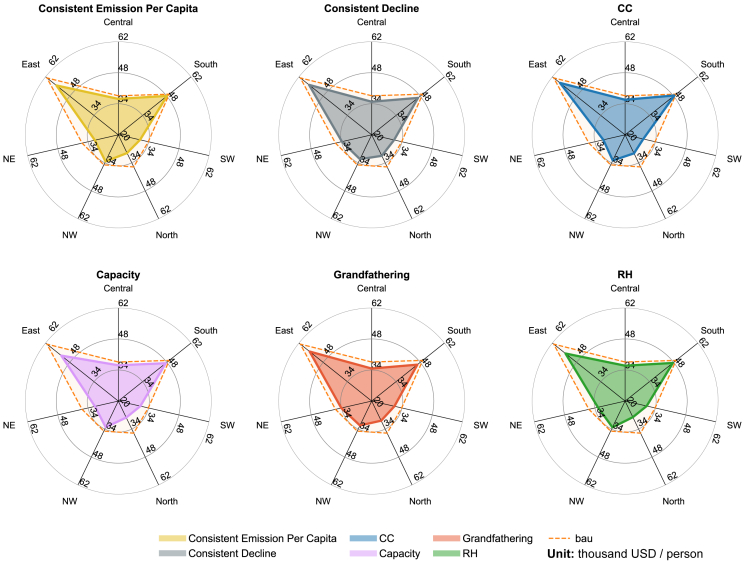
Regional GDP per capita in 2060.

An analysis of the regional level reveals obvious patterns. The three northern regions (NW, N, and NE) suffer more than other regions (Figure 3). The equity-based scenarios exhibit a capacity to mitigate the losses experienced by the most Northern provinces, though it does not fundamentally alter the overall national economic landscape. However, it is imperative to note that some provinces stand out as unique exceptions in this context. These provinces either have very limited or no coal-related industries, resulting in an increase in per capita GDP under efficiency-based policies, such as Hainan (S) and Tibet (SW). Alternatively, due to equity-based policies granting relatively less-developed regions more emissions budgets to promote economic development, per capita GDP increases under such policies. Such phenomenon can be found in Yunnan (SW) and Guangxi (S).

**Figure 4:**
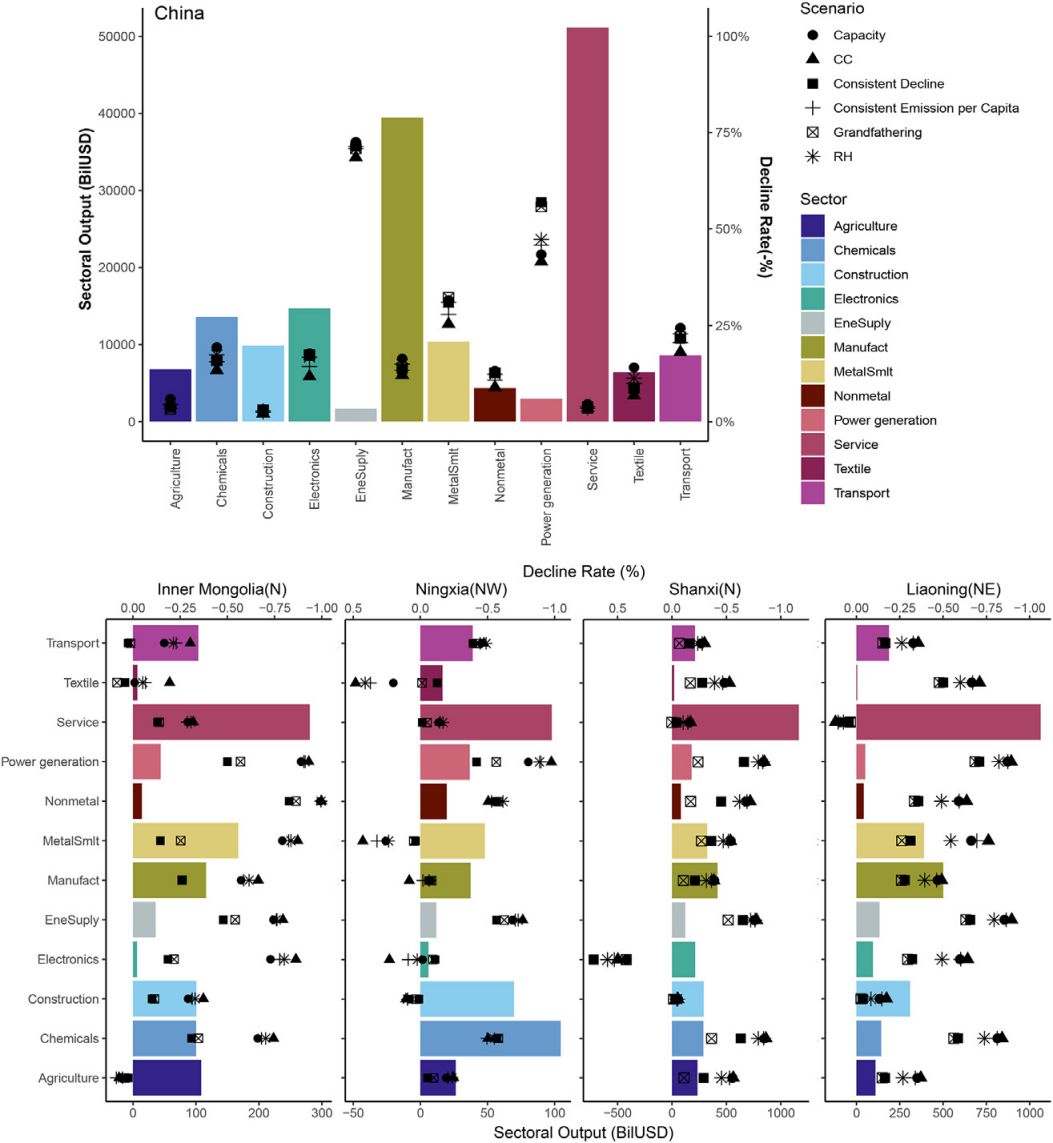
Sectoral output and comparative reduction rate in 2060 for China and four coal-reliant provinces (in which bars stand for the BaU scenario, while points denote different policy scenarios).

The simulation results (Figure 4) show that in 2060, the industrial structure relies more on low-carbon and low-coal sectors, such as the service sector, and less on traditional energy industries, including power generation and energy supply sectors. However, the overall sectoral output decreases due to carbon neutrality policies. The output of service sector reaches 51,120 BilUSD under the BaU scenario in 2060, accounting for 30% of the total, followed by manufacturing sector, which accounts for 23%. The absolute value of the sectoral output of the service sector decreases with policy interventions, but its share percentage increases. For the two energy supply industries, which are also the major coal consumption sectors, their output shares will decrease significantly. Under the CC scenario, the output of energy supply sector is approximately 69% lower than that without policy intervention, and it increases to 71% under the RH scenario.

To focus explicitly on the provinces with the highest coal reliance, we have selected the top four provinces with the greatest coal intensity in 2060 under the BaU scenario. These provinces are Inner Mongolia (N), Ningxia (NW), Shanxi (N), and Liaoning (NE). The impact of efficiency-based policies on the sectoral output of these four provinces tends to have a more substantial effect on the sectoral output of these provinces. Among the four provinces, in the BaU scenario, the 2060 total sectoral output for Inner Mongolia and Ningxia is 1,084 BilUSD and 514 BilUSD, respectively, whereas Shanxi and Liaoning both exceed 3,000 BilUSD in the total sectoral output. It is noteworthy that many sectors within all four provinces experience sever decline in output due to policy interventions. The overall negative impacts are more pronounced when compared to the national level, and in some cases, certain sectors may face the risk of being forced out of the provincial economy. For instance, the non-metal smelting sector in Inner Mongolia and the power generation sector in Ningxia may be particularly vulnerable. Although we do note marginal positive effects on the textile and transport sectors of Inner Mongolia from the grandfathering and consistent decline scenario, it is evident that carbon neutrality policies alone may not be sufficient to facilitate the transformation of Inner Mongolia’s economic structure toward a low-carbon and sustainable model. Despite the fact that all four provinces experience substantial negative impacts from policy interventions, the other three, Ningxia, Shanxi, and Liaoning, are relatively better off, as certain sectors show signs of growth under the policy scenarios. To illustrate, in Ningxia, carbon neutrality policies result in increased output across four sectors. In Shanxi, the electronics sector experiences substantial growth, with output increasing by 50% in the CC scenario and 59% in the RH scenario. Furthermore, these policies boost the output value of Liaoning’s service sector, contribute to its long-term sustainability.

### Northward embodied coal and coal leakage driven by equity-based policies

**Figure 5:**
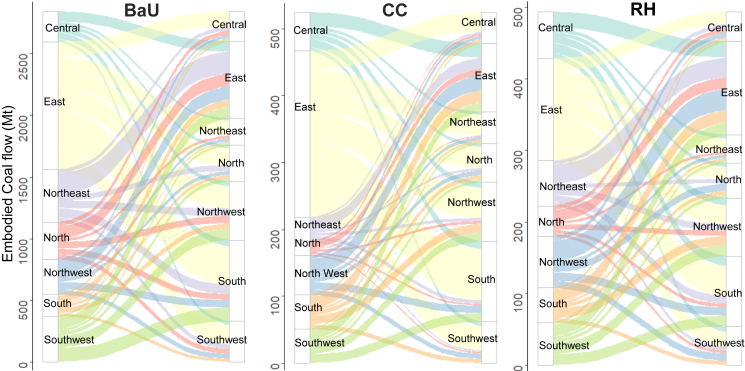
Sankey diagram for embodied coal consumption with provincial bilateral trade under the Three Scenarios by 2060 (in which the regions on the left y axis are the producers of the products that are eventually consumed by the provinces on the right y axis, and the flows are the amount of embodied coal consumption.).

The total embodied coal consumption associated with provincial bilateral trade, hereinafter termed embodied coal consumption, declines by approximately 80% in the policy scenarios, which means that the carbon neutrality policy reduces not only coal intensity nationwide but also the embodied coal consumption in China. Furthermore, the uneven regional embodied coal consumption is alleviated in the RH scenario. Figure 5 shows a Sankey diagram for regional embodied coal consumption. In the BaU and CC scenarios, the geographic pattern is similar, where the eastern region is the largest producer of coal products. This phenomenon becomes even more evident in the CC scenario, in which the eastern region alone is responsible for almost half (47%) of the embodied coal consumption, and the southern region is the main consumer of coal-related products produced in the eastern region. However, if policymakers provide the relatively less-developed provinces with a more carbon budget and transfer the burden to the relatively well-developed provinces like eastern and southern region, as assumed in the RH scenario, the share of the eastern regional embodied coal consumption will be cut to 27%. In addition, the share of three northern regions accounts for only 22% in the CC scenario, but this increases to 34% in the RH scenario. This means that the proposed carbon neutrality policies significantly impact local industrial structures through domestic trade. When less-developed regions, mainly located in three northern regions, are favored with moderate carbon intensity constraints, more coal-reliant products will be produced northward. This phenomenon, therefore, alleviates GDP losses in Northern China, as presented in Figure 3.

The previous results show that both efficiency-based and equity-based policies significantly affect embodied coal consumption reduction. In order to conduct a thorough examination of the factors influencing changes in embodied coal consumption within the industrial sector and along the related supply chain, we employ the SPD method. Due to the inherent limitations in the SPD method, such as the constraints on interpreting supply,^29^ we focus our analysis on the most crucial supply chains within China.

**Figure 6:**
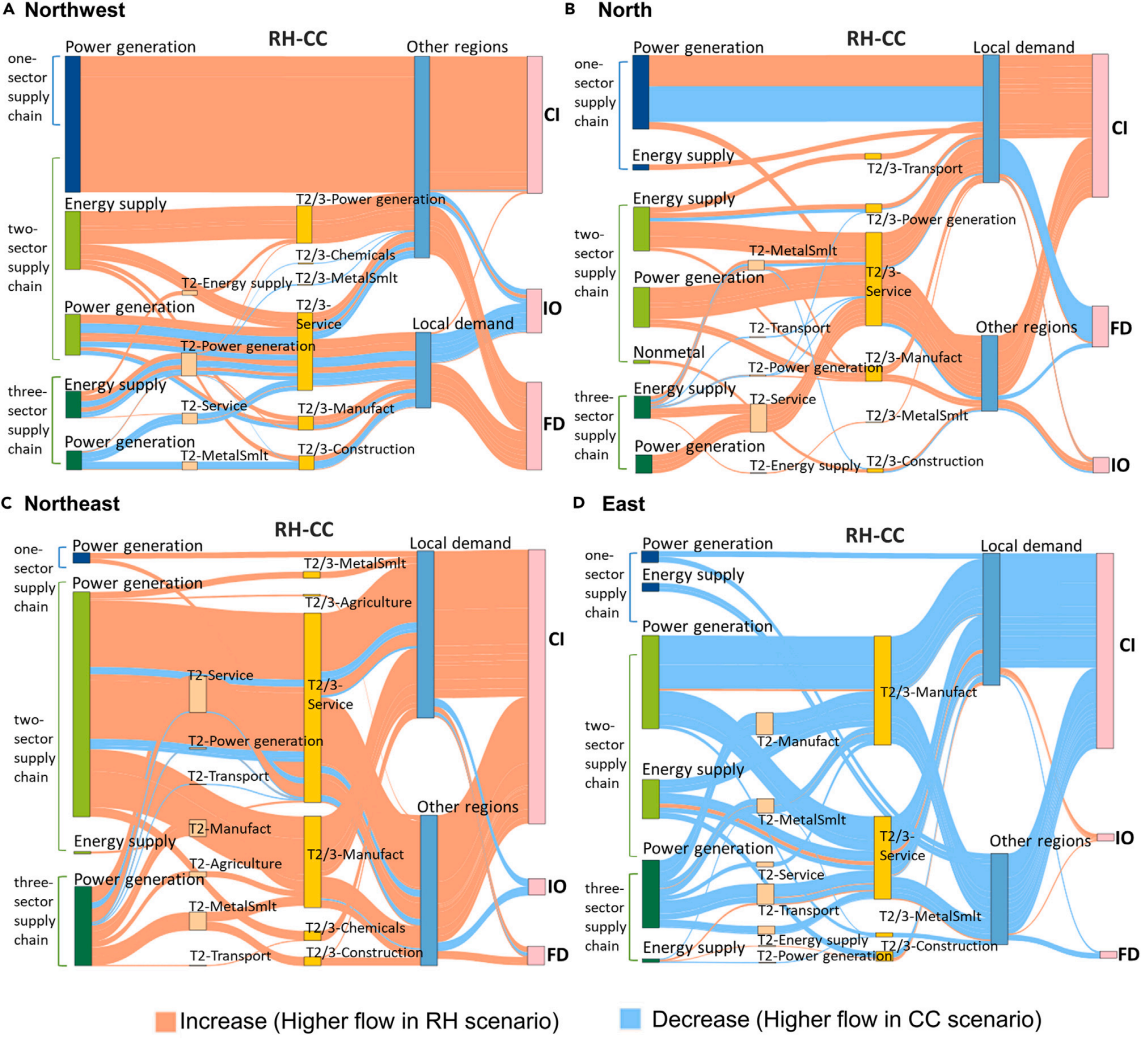
Illustrative map of differences in embodied coal consumption flow between the RH and CC scenarios for 4 typical regions in 2060 (A. Northwest; B. North; C. Northeast; D. East. The nodes on the left side are the supply sectors, and the different colors distinguish supply chains with different numbers of sectors) The numbers represent the industry’s position in the supply chain. The sectors at the end of the supply chain are the final demand sectors. CI, IO, and FD represent the coal intensity effect, input-output coefficients change effect, and final demand effect, respectively.

According to the SPD results, the most crucial embodied coal consumption reduction factor is the reduction of coal intensity in both CC and RH scenario. More specifically, coal intensity reduction in the energy supply industries is vital for the entire supply chain. The service, manufacturing, and construction sectors are these supply chains’ most important demand sectors. This universal pattern is found in all regions. Figure 6 shows the changes in embodied coal consumption flows between the CC and RH scenarios in four typical regions, which are the three northern regions and eastern region. It is noted that when carbon intensity constraints become tougher for well-developed regions, as suggested in most equity-based scenarios, there is a discernible increase in embodied coal consumption in the power generation sector in less-developed regions. The burden of embodied coal consumption is the largest in the northwest region. In the RH scenario, the total embodied coal consumption in the northwest region is 40 Mt higher than that in the CC scenario, and 45% (18 Mt) of which is because of the increasing final demand for electricity from other regions. A similar pattern can also be found in the central and southwest regions (See Appendix). However, this phenomenon is less evident in northern region (Figure 6B), probably because two out of five provinces in northern region, Beijing and Tianjin, are well-developed municipalities. Furthermore, Figure 6D shows that embodied coal consumption decreases significantly in the eastern region, especially power generation for local demand. The decrease in embodied coal consumption in the local power generation-manufacture chain alone goes up to 16 Mt, which is 42% of the total coal reduction in the power generation sector. These conclusions suggest that the alleviated GDP loss in the less-developed provinces and the additional coal intensity reduction in the well-developed provinces in the RH scenario may come from the demand transfer of the power generation sector.

## Discussion

The comprehension of coal decoupling in the transition toward carbon neutrality holds significant implications for the sustainable development of both the national economy and the environment in a synergistic manner. The results indicate that implementation of carbon neutrality policies will lead to a reduction in coal intensity to approximately half of its 2017 level by around 2030. Coal intensity declines by 96% to merely 7 ktoe/BilUSD when the carbon constraints are the same for all provinces, leaving the rest as backup energy for emergency response such as peak load shaving. This finding is consistent with the operational guidance on energy that coal-fired power should still be the backup energy supporting peak power demand under extreme circumstances.^30^ In other words, if no specific policy is tailored to coal-fired electricity, energy supply, and power generation could be among the last to witness significant reductions in coal consumption. Furthermore, the national GDP loss in 2060 reaches 4,951 BilUSD in efficiency scenarios and 5,643 BilUSD in equity scenarios. The substantial economic losses may hinder the progress of coal decoupling and carbon neutrality policies, especially in Northern China. Its absolute coal intensity reduction is far beyond that of the southern region, especially the southeast coastal provinces, which are relatively well developed. The geographic pattern of coal-reliant provinces primarily located in northern regions is driven by coal-coupled economic development and a carbon-intensive industrial structure. Three northern regions often have a historical reliance on coal as a cheap source of energy and a significant presence of industries that are highly carbon-intensive, such as heavy manufacturing and energy production.

From the sectoral perspective, most sectors nationwide will phase down coal consumption by 2060 under the carbon neutrality target, and the Chinese industrial structure relies more on low-coal sectors. This is also consistent with the findings of Jia et al. (2022),^31^ who concluded that after 2015, economic development gradually decoupled from coal consumption in China, and its marginal benefits decreased as the share of tertiary industry increased. When relatively well-developed provinces are required to carry more carbon reduction burdens (equity-based policies), coal-reliant production, especially those in the energy supply and power generation sectors, is relocated northward. As a result, the demand for electricity production decreases in the well-developed regions, but increases in less-developed three northern regions, namely northwestern, northern, and northeastern regions.

The allocation of regional carbon intensity or cap targets could be regarded as a more flexible instrument to regulate energy structure optimization, giving local entities a better ability to adjust their energy mix at lower transition costs. However, although it does not contravene the national carbon neutrality target, policies that favor relatively less-developed regions based on the ability-to-pay principle may eventually increase the coal intensity compared to those under efficiency-based scenarios, as well as the embodied coal consumption and carbon leakage associated with provincial bilateral trade. On one hand, such relocation helps provinces in three northern regions with GDP growth, alleviating GDP losses caused by carbon constraints. On the other hand, it is difficult to argue whether increased consumption of coal in those regions for higher GDP development is considerable or not, as the air pollution and health impacts brought by burning coal cannot be neglected. Therefore, end-of-pipe technology is essential to remove harmful substances. In addition, we suggest that if the government wants to alleviate the uneven economic burden among provinces, except for putting stricter carbon intensity on the well-developed provinces, policymakers should keep investing in cleaner energy to help the relatively less-developed provinces drive out coal smoothly. The government should keep encouraging the development of negative emission technologies (NETs), as these provide carbon emission space. If the development of NETs does not occur as we assume in this paper, then the coal intensity criteria should be further tightened correspondingly, leading to even higher macroeconomic impacts.

### Limitations of the study

Finally, we conclude by pointing out some caveats. Here, we discuss mainly the provincial and sectoral coal intensity under the carbon neutrality goal in China, three extensions could be explored in future research. First, our current model does not explicitly account for or project the endogenous emergence of groundbreaking low-carbon technologies and industries catalyzed by the transformative shift toward carbon neutrality. Our study primarily focuses on the imminent challenges confronted by traditional industries, which could potentially result in an overestimation of macroeconomic losses and a relatively conservative estimate of future economic resilience. Second, our analysis does not take into account the international carbon leakage of carbon neutrality. Such effects could spread to the domestic energy market, and should be further studied.

## STAR★Methods

### Key resources table

**Table:** 

REAGENT or RESOURCE	SOURCE	IDENTIFIER
**Deposited data**		

National-level energy consumption	Statistical Review of World Energy	https://www.bp.com/content/dam/bp/business-sites/en/global/corporate/xlsx/energy-economics/statistical-review/bp-stats-review-2022-all-data.xlsx
Provincial-level Energy Balance Table	China Energy Statistical Yearbook	http://www.stats.gov.cn/tjsj/tjcbw/
Provincial-level renewable energy production	China Electric Power Yearbook	http://www.stats.gov.cn/tjsj/tjcbw/
Input-output table	National Bureau of Statistics of China (NBS)	Input-output Tables of China

**Software and algorithms**		

IMED|CGE	IMED|CGE	https://www.imedmodel.com/
R-4.0.0	R	https://www.r-project.org/; RRID: SCR_001905
Python 3.9.12	Python	https://www.python.org/

### Resource availability

#### Lead contact

Further information and requests for resources and reagents should be directed to the corresponding author (l.zhang@cityu.edu.hk).

#### Materials availability

This study did not generate new unique materials.

#### Data and code availability

Data: All data reported in this paper will be shared by the lead contact upon request.

Code: All custom codes can be available on request from the lead contact.

Any additional information required to reanalyze the data reported in this paper is available from the lead contact upon request.

### Method details

#### IMED|CGE model

In order to measure the coal reduction target in the context of carbon neutrality, and assess its various impacts on China, we use the Integrated Model of Energy, Environment and Economy for Sustainable Development | Computable General Equilibrium model (IMED|CGE) to simulate pathways for the period 2017–2060. The IMED|CGE model is a recursive-dynamic one-year step CGE model, one of the few Integrated Assessment Models (IAMs) developed by Chinese scholars that is flexible for various research objectives. The model has been widely used in environmental economics-related studies to evaluate low-carbon policies.^28^^,^^32^^,^^33^^,^^34^

The IMED|CGE model represents the economies of 31 Chinese provinces (excluding Taiwan, Hong Kong, and Macao due to lack of data) and 25 sectors. There are four blocks interacting with each other simultaneously in the model, which are the production block, the market block covering domestic and international transactions, and the blocks covering the income and expenditure of the government and household, as presented in Figure S10.

In line with neoclassical economic theory, the model solves for the optimized level of prices at equilibrium in interrelated sectors under a set of constraints. In this study, one of the most important constraints is the carbon emission cap imposed for each province. Under the assumption of market equilibrium, the price of a commodity within a given province is influenced by three key factors: the local supply within that province, the distribution to domestic markets in other provinces, and the export to international markets. The imposition of an emission cap restricts the utilization of primary energy, which is an intermediate input, leading to reductions in commodity prices.

We employ Leontief functions to simulate the physical thermal laws governing the interaction between carbon emissions and various fossil fuel combustion processes, and apply multi-level nested constant elasticity substitution (CES) functions to simulate production activities. Consequently, when carbon emissions are affected by the carbon policy, energy input changes accordingly, leading to corresponding adjustments in the equilibrium outcomes. We illustrate the impact of the carbon policy on the production block within the CGE model in Figure S11, shedding light on the primary factors contributing to the reduction in output.

Determination of the numeric assumptions for the characteristics and preferences of economic agents decides the reliability of the models. The initial input data in the IMED|CGE model are quoted from the 2017 National Input-Output Table (IO table)^35^ and Energy Balance Table (EBT),^36^ which reflect the comprehensive Chinese economic and energy situation. To ensure the consistency of the table format, the energy consumption data from the EBT table are converted into the IO table’s order. Furthermore, to better interpret the results, the 25 sectors from the CGE model are further integrated into 12 sectors in the post-analysis, and the convention table is presented in Table A1. In addition, for the period 2018–2021, the ex-post model simulation has been adjusted to be consistent with the real data, improving the *ex ante* reliability for the 2022–2060 period.

#### Scenario design

Emissions of carbon dioxide (CO_2_) in 2060 are regarded as the indicator of the success of the carbon neutrality target, so the CO_2_ emission targets for the 31 provinces and the whole nation are significant. The literature shows that bioenergy with carbon capture and storage (BECCS)^13^^,^^37^^,^^38^ and carbon sinks^39^^,^^40^ together can provide China with an approximately 0.68–2.10 Gigaton (Gt) CO_2_ emission budget nationwide in 2060. Therefore, in this paper, we employ the positive estimate of 2.10 Gt CO_2_ as the nationwide emission target for China in 2060. Furthermore, we consider future technology improvement as investment in the model.

To test to what extent the carbon neutrality goal can help phase down coal consumption, one baseline scenario and six policy scenarios are designed to measure the impacts of various climate policies on carbon neutrality. Business-*as*-Usual (BaU) scenario, which adheres to the current climate policies in China, including the National Determined Contributions (NDCs). It is assumed that the current policies will continue without political disruptions. The future projections of GDP and population in this baseline scenario follow the estimations under the Shared Socioeconomic Pathway 2 (SSP2) scenario.^41^^,^^42^ No carbon constraint is employed in the BaU scenario.

To incorporate more comprehensive policy interventions and avoid making unwarranted assumptions that could lead to non-robust results, we have established six policy scenarios. In general, these scenarios align with either the principle of efficiency or the principle of equity. Brief descriptions of these scenarios can be found in scenario table below, while the corresponding formulas are provided in the supplemental materials.

In the main text, we focus on the results of two carbon neutrality policy scenarios: the Consistent Command (CC) and Robin Hood (RH) scenarios. These scenarios represent two different basic principles. Details of the remaining scenarios can be found in the appendices. The selection of these two scenarios is based on their alignment with either the most probable policy interventions in China, rooted in past experiences, or a scenario designed to demonstrate potential policy advantages for less-developed regions, in line with China’s anti-poverty initiatives.

#### Scenario Table

**Table:** 

Basic Rule	Scenario	Description	Principal
Business as usual	BaU		
**Efficiency-based**	**Consistent Command (CC)**	Requiring all provinces to achieve the same per GDP emission in 2060.	Efficiency principle.
	**Consistent Decline**	Requiring all provinces to reduce their emission with the same yearly decline rate.	Sovereignty principle.
**Equity-based**	**Consistent Emission per Capita**	Requiring all provinces to achieve the same per capita emission in 2060	Equity allocation principle.
	**Robin Hood (RH)**	Provinces with a higher per capita GDP in 2020 will be allocated a diminished quantity of carbon permits.	Ability-to-Pay allocation principle.
	**Grandfathering**	Provinces that have a greater historical accumulation of emissions will be allocated a larger number of carbon permits.	Grandfathering allocation principle.
	**Capacity**	Regions with a higher per capita GDP in 2060 will be allocated a diminished quantity of carbon permits.	Ability-to-Pay allocation principle.

The specific emissions of each province under different scenarios are shown in supplemental materials.

#### Environmentally extended input-output analysis

Environmentally extended input-output analysis (EIOA) has gained widespread acceptance as a valuable tool for elucidating the holistic environmental reactions triggered by economic activities. In this paper, the results simulated from IMED|CGE model serve as pivotal inputs in EIOA. EIOA is used to quantify coal consumption embodied in domestic bilateral trade, which helps determine and analyze the interconnection of sectors in different regions.^43^ Detailed formulas underpinning these calculations are available in the appendices for reference.

For each province, the monetary balance is

(Equation 1) $$\begin{array}{c} X^{p}=Z^{p}+y^{p}+\sum_{s}e^{ps} \end{array}$$

where $X^{p}$ is a vector for the total sectoral outputs in province *p*. To avoid infinite computation due to the interconnection of sectors, only the products produced in province *p* are considered. $Z^{p}$ represents the domestic and imported industry requirements, and $y^{p}$ is the final demand (household, government, and investment) of domestic and imported products in that province; $e^{ps}$ is the imports to province *s* from province *p*.

The total direct and indirect coal consumption for province *p* to produce products that are eventually consumed in province *s* is

(Equation 2) $$\begin{array}{c} T^{ps}=F^{p}{\left(I-A^{pp}\right)}^{-1}e^{ps} \end{array}$$

where $F^{p}$ is the direct coal intensity in province *p*. $L={\left(I-A\right)}^{-1}$ is the Leontief inverse matrix, which captures both direct and indirect inputs to satisfy one unit of final demand in monetary value. Again, only the domestic supply chain is considered.

#### Structural path decomposition analysis (SPD)

To comprehensively investigate the determinants of coal consumption changes within the industrial sector and across the associated supply chain, we employ the structural path decomposition (SPD) method, a novel research methodology that integrates structural decomposition analysis (SDA) and structural path analysis (SPA).^44^ While SDA offers a robust means of dissecting the underlying drivers of coal consumption alterations, it inherently lacks the capacity to elucidate these impacts at the supply chain level. Conversely, SPA is a valuable tool for assessing the effects within a supply chain but falls short in identifying the pivotal factors influencing coal consumption changes within that chain.^29^ By employing SPD, we aim to unveil the intricate dynamics of coal consumption changes across various scenarios and to pinpoint the salient factors responsible for these fluctuations.

To distinguish the factors that change simultaneously, the change in coal consumption d(E) is decomposed into coal consumption intensity change (F), intermediate input structure change (L), and final demand change (y):

(Equation 3) $$\begin{array}{c} d\left(E\right)=d\left(F\right)Ly+Fd\left(L\right)y+FL\left(dy\right) \end{array}$$

In addition, we adopt the average weighted decomposition method, which can be expressed as follows:

(Equation 4) $$\begin{array}{c} △E_{F}=\frac{1}{3}*△FL_{0}y_{1}+\frac{1}{6}*△FL_{1}y_{0}+\frac{1}{6}*△FL_{0}y_{1}+\frac{1}{3}*△FL_{1}y_{1} \end{array}$$

(Equation 5) $$\begin{array}{c} △E_{L}=\frac{1}{3}*F_{0}△Ly_{0}+\frac{1}{6}*F_{0}△Ly_{1}+\frac{1}{6}*F_{1}△Ly_{0}+\frac{1}{3}*F_{1}△Ly_{1} \end{array}$$

(Equation 6) $$\begin{array}{c} △E_{y}=\frac{1}{3}*F_{0}L_{0}△y+\frac{1}{6}*F_{0}L_{1}△y+\frac{1}{6}*F_{1}L_{0}△y+\frac{1}{3}*F_{1}L_{1}△y \end{array}$$

#### Indicators of coal reduction

In the paper, we opted for 'coal intensity' instead of 'coal consumption' because large coal consumption can be a result of substantial production rather than a significant need for coal. This approach allows for a more equitable comparison among provinces with varying production sizes and resource endowments. Coal intensity is calculated as follows:

(Equation 7) $$\begin{array}{c} {Coal\;Intensity}_{P}=\frac{{Coal\;Consumption}_{P}}{GDP} \end{array}$$

Except for the base year, coal consumption and output data for all year under examination are derived from the IMED|CGE model. Furthermore, in order to gain a comprehensive understanding of coal dependence, we extend the analysis beyond regional considerations and delve into 12 aggregated sectors in the 31 provinces, as outlined below:

(Equation 8) $$\begin{array}{c} {Coal\;Intensity}_{s}=\frac{{Coal\;Consumption}_{s}}{{Sectoral\;Ouput}_{s}} \end{array}$$

where *s* stands for sector. To ensure accurate measurement of sectoral coal intensity, we employ sectoral output values in the equation rather than relying on value added. This approach helps avoid potential biases, especially when assessing coal intensity in certain industrial sectors, such as energy supply and power generation. Given that the value added in these sectors as primary products may be significantly lower compared to other sectors, employing it in the calculation could lead to an overestimation of their coal intensity.

Jenks natural breaks classification is a statistical technique often used in Geographic Information Systems. It aims to identify “natural” groupings within a dataset, arranging data into classes where the values are naturally clustered together. This method ensures the class breaks are defined in a manner that optimally groups similar values and maximizes the distinctions between classes. The classification was performed using Jenkspy (version 1.23.5) package in Python software. Please check Table S3 for break points.

In addition, to determine whether coal intensity follows a decreasing trend during the simulated period 2017–2060, we employ the Mann-Kendall trend test, which is a commonly used tools for detecting discernible trends in time series.^45^ The null hypothesis of the Mann-Kendall test is that no specific monotonic trend in the series is found, while the alternative is that a specific monotonic trend exists, either positive, negative, or neutral.
