# Supplementary material for: Equity-based carbon neutral plan induces cross-regional coal leakage and industrial relocation
Source: iScience. 2024 Feb 1;27(3):109079. doi: 10.1016/j.isci.2024.109079 (PMC10867442; doi:10.1016/j.isci.2024.109079)
Supplement: Document S1. Figures S1–S11 and Tables S1–S3 [file mmc1.pdf]

iScience, Volume *27*

Supplemental information

Equity-based carbon neutral plan induces cross-regional coal leakage and industrial relocation

Ziqiao Zhou, Xiaotian Ma, Silu Zhang, Chaoyi Guo, Xiaorui Liu, Lin Zhang, and Yang Xie

Supplemental Items

Formula for Policy Scenarios

1. **Consistent Command(CC)**

$$E_{i,2060} = \frac{E_{China,2060}}{GDP_{China,2060}} = 0.036\ \ tCO_2/Million\ \ USD \tag{1}$$

$E_{i,2060}$ is the carbon emission permits for province i in 2060, and $E_{China,2060}$ is the national carbon emission permits in 2060. CC requires all provinces to achieve the same per GDP emission in 2060. National emission budget is projected to be 2.1Gt, as stated in the main text. The $GDP_{China,2060}$ is estimated under the BaU scenario. Smooth linear projection is used to calculate the emission trajectory from 2018 to 2059.

2. **Consistent Decline**

$$E_{i,t} = E_{i,t-1} * R_{China,t} \tag{2}$$

$E_{i,t}$ is the carbon emission permits for province i in year t, and $R_{i,t}$ is the carbon emission decline rate in year t. This scenario requires all provinces to reduce their emission with the same yearly decline rate. The emissions are set to peak in around 2025 and then decline by -1% to -7% yearly rate.

3. **Consistent Emission Per Capita**

$$E_{i,2060} = E_{China,2060} * \frac{pop_{i,2060}}{\sum_{i=1}^{31} pop_{i,2060}} \tag{3}$$

$E_{i,2060}$ is the carbon emission permits for province i in 2060, and $E_{China,2060}$ is the national carbon emission permits in 2060. $pop_{i,2060}$ stands for population in province i in 2060. This scenario requires all provinces to achieve the same per capita emission in 2060.

4. **Robin Hood(RH)**

$$E_{i,2060} = E_{China,2060} * \frac{pop_{i,2020} * \left(GDPPC_{i,2020}\right)^{-\alpha}}{\sum_{i=1}^{31}\left[pop_{i,2020} * \left(GDPPC_{i,2020}\right)^{-\alpha}\right]} \tag{4}$$

RH scenario allocates provinces that have a higher per capita GDP in 2020 a diminished quantity of carbon permits. The variable α allows different magnitudes of change for the allocation in response to changes in GDP per capita. If α is less than 1, the change in allocation is less than that in GDP per capita. In this paper, we examine the extreme situation where α equals 1, meaning that the elasticity is the same for both the allocation and GDP per capita.

5. Grandfathering

$$E_{i,2060} = E_{\text{China},2060} \times \frac{\sum_{1997}^{2019} E_{i,t}}{\sum_{i=1}^{31} \sum_{1997}^{2019} E_{i,t}} \tag{5}$$

This scenario allocates provinces that have a greater historical accumulation of emissions a larger number of carbon permits. Due to data availability, emission data of Tibet is estimated based on the national emission growth rate from 1997 to 2019.

6. Capacity

$$E_{i,2060} = E_{China,2060} * \frac{pop_{i,2060} * \left(GDPPC_{i,2060}\right)^{-\alpha}}{\sum_{P=1}^{31}\left[pop_{i,2060} * \left(GDPPC_{i,2060}\right)^{-\alpha}\right]} \tag{6}$$

This scenario allocates regions that have a higher per capita GDP in 2060 a diminished quantity of carbon permits.

Table S1. Aggregated Sector List

Aggregated Sectors	Original Sectors
Energy Supply	Coal Mining, Natural Gas, Other Mining, Petrol Oil, Manufactured Gas
Power Generation	Power Generation
Agriculture	Agriculture
Chemicals	Chemicals
Construction	Construction
Electronics	Electronics
Manufacture	Textile, Paper, Food Production, Machinery, Other Manufacturing,
Metal Smelting	Metal Smelting
Non-Metal Smelting	Non-Metal Smelting

Service	Service, Water Supply
Transportation	Transportation, Transportation Equipment

Table S2. Geographic Regions

Geographic Regions	Provinces	Notes
East (**E**)	Shandong, Jiangsu, Anhui, Shanghai, Zhejiang, Jiangxi, Fujian	Affiliated with the southeast costal region.
South (**S**)	Guangdong, Guangxi, Hainan	Affiliated with the southeast costal region.
Southwest (**SW**)	Sichuan, Guizhou, Yunnan, Chongqing, Tibet	
Centre (**C**)	Henan, Hubei, Hunan	
Northeast (**NE**)	Heilongjiang, Jilin, Liaoning	Affiliated with Northern China
North (**N**)	Beijing, Tianjin, Hebei, Shanxi, Inner Mongolia	Affiliated with Northern China
Northwest (**NW**)	Shaanxi, Gansu, Ningxia, Qinghai, Xinjiang	Affiliated with Northern China

Table S3. Jenks Natural Break Point

Object	Break Points	Notes
Regional Coal Intensity	[0, 158.5], [158.5, 288.8], [288.8, 552.1], [552.1, 856.9], [856.9, 1126.8]	Class = 5
Sectoral Coal Intensity	[0, 125.7], [125.7, 705.6], [705.6, 1289.8], [1289.8, 2014.0], [2014.0, 3400.8]	Class = 5

Note: Jenkspy (version 1.23.5) package in Python

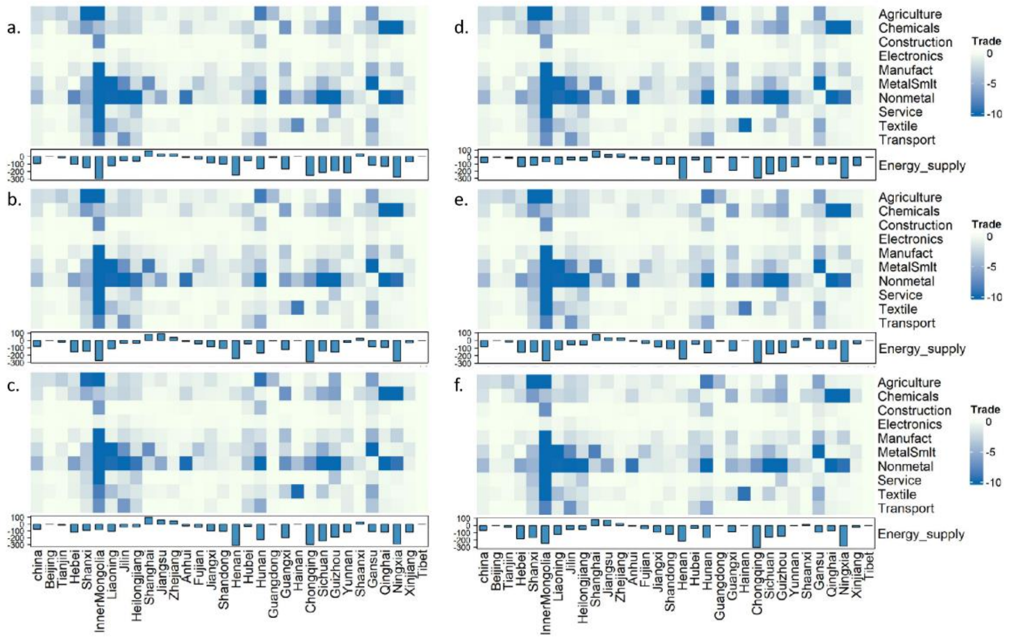

Fig. S1 Sectoral Coal Intensity of 31 provinces in 2060 (a. Consistent Command(CC), b. Robin Hood(RH), c. Grandfathering, d. Consistent Decline, e. Consistent Emission per Capita, f. Capacity)

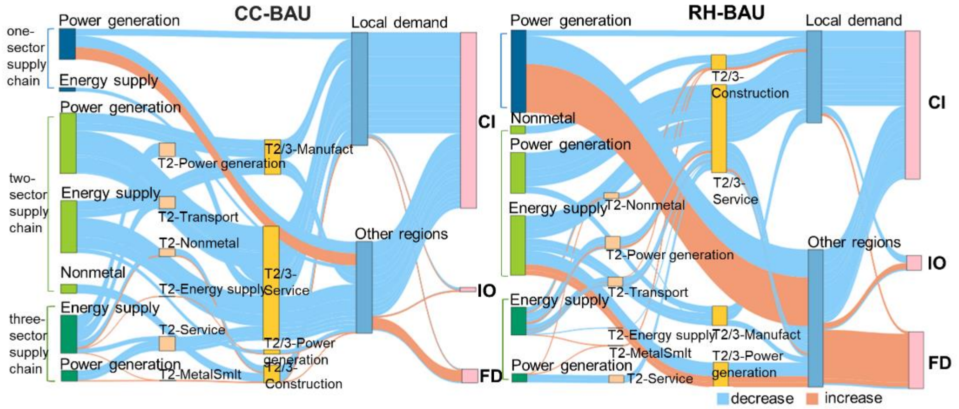

Fig. S2 Illustrative Map of Embodied Coal Consumption Flow Changes in Northeast Region in 2060

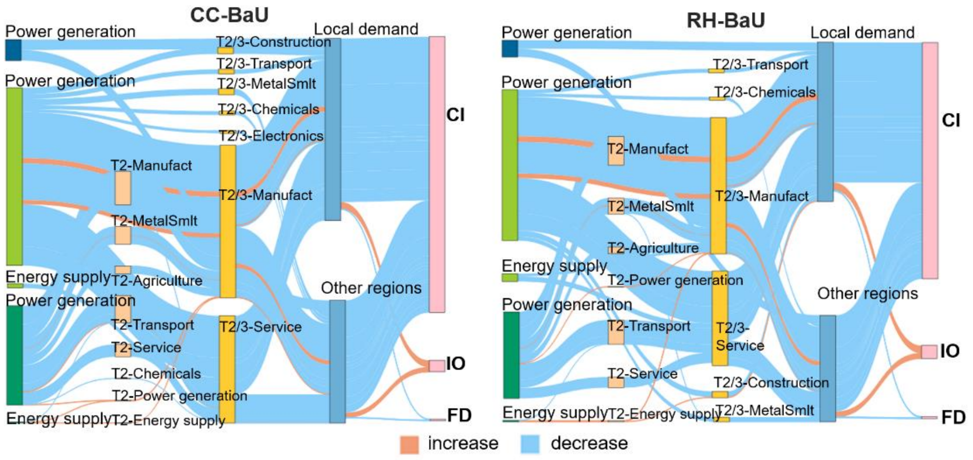

Fig. S3 Illustrative Map of Embodied Coal Consumption Flow Changes in East Region in 2060

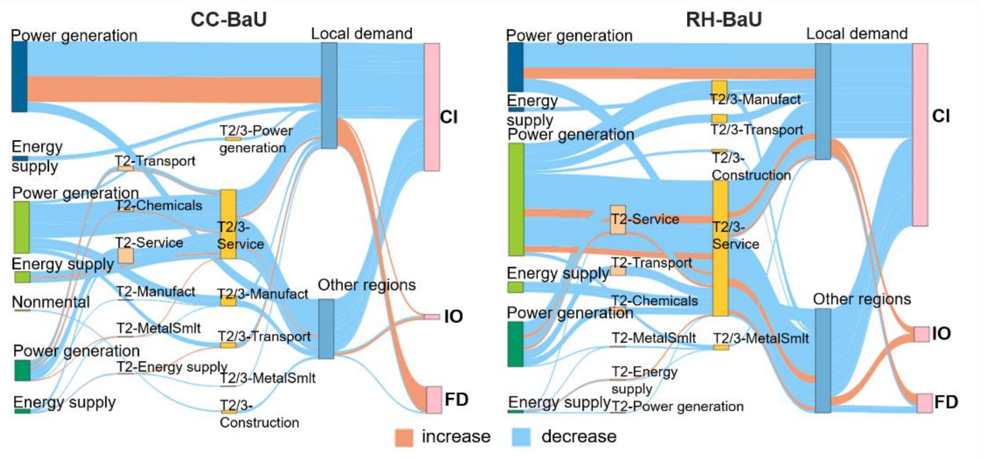

Fig. S4 Illustrative Map of Embodied Coal Consumption Flow Changes in North Region in 2060

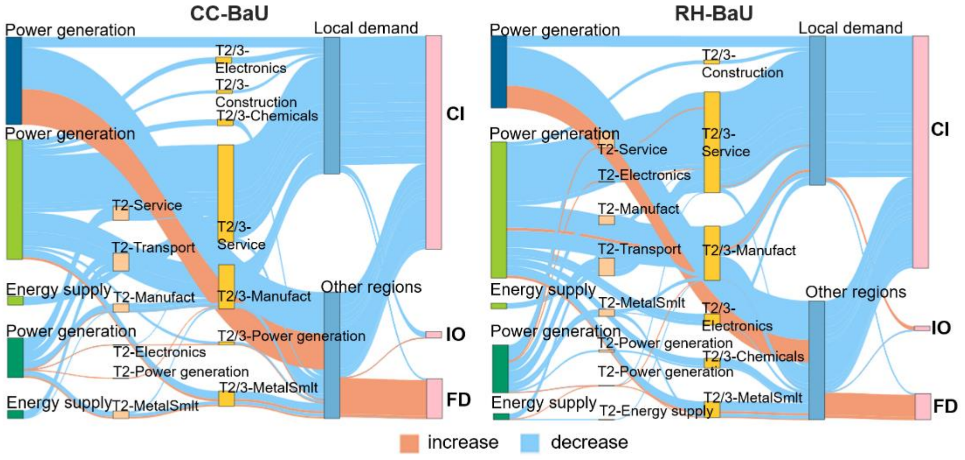

Fig. S5 Illustrative Map of Embodied Coal Consumption Flow Changes in South Region in 2060

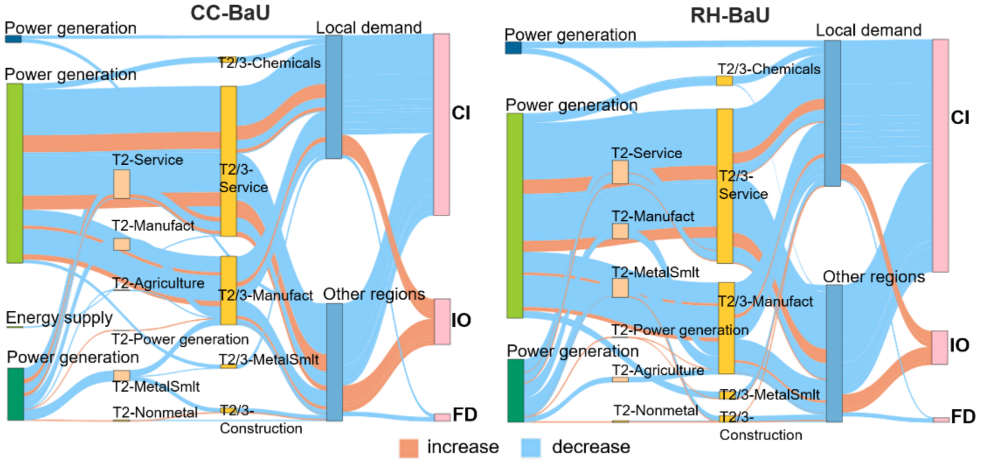

Fig. S6 Illustrative Map of Embodied Coal Consumption Flow Changes in Northeast Region in 2060

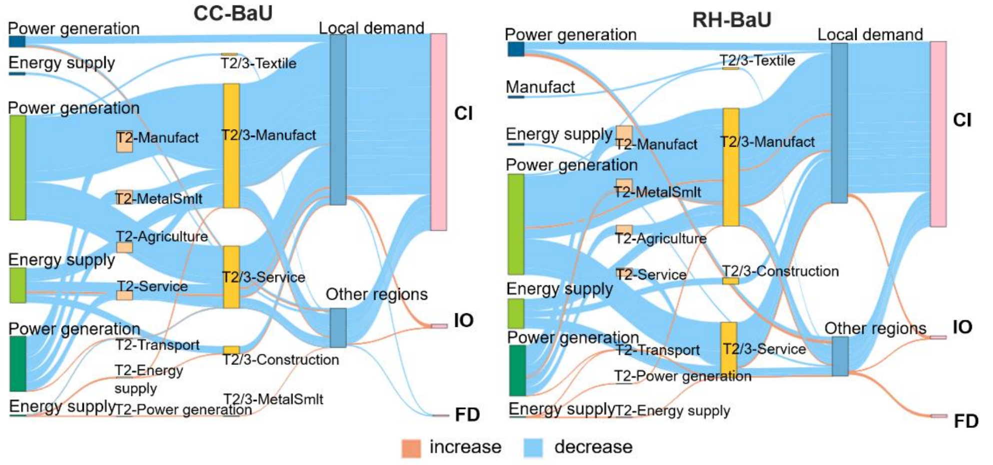

Fig. S7 Illustrative Map of Embodied Coal Consumption Flow Changes in Central Region in 2060

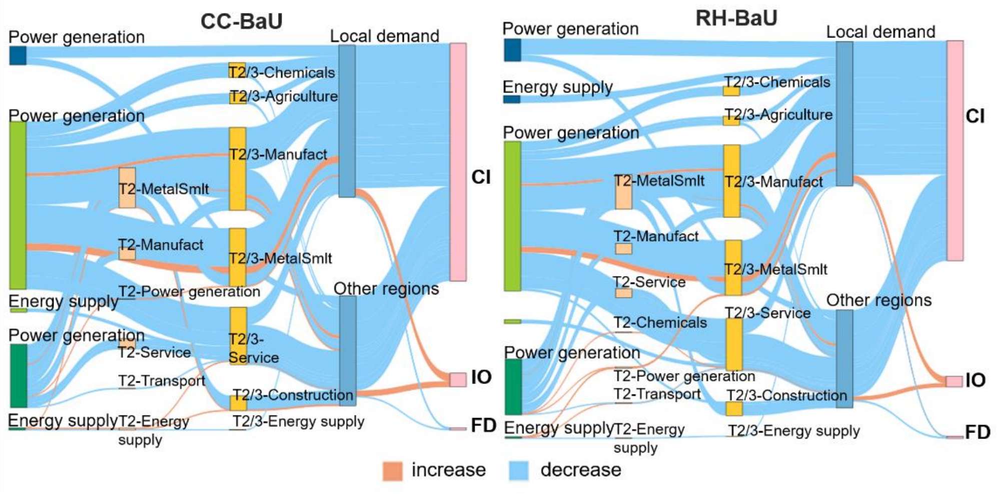

Fig. S8 Illustrative Map of Embodied Coal Consumption Flow Changes in Southwest Region in 2060

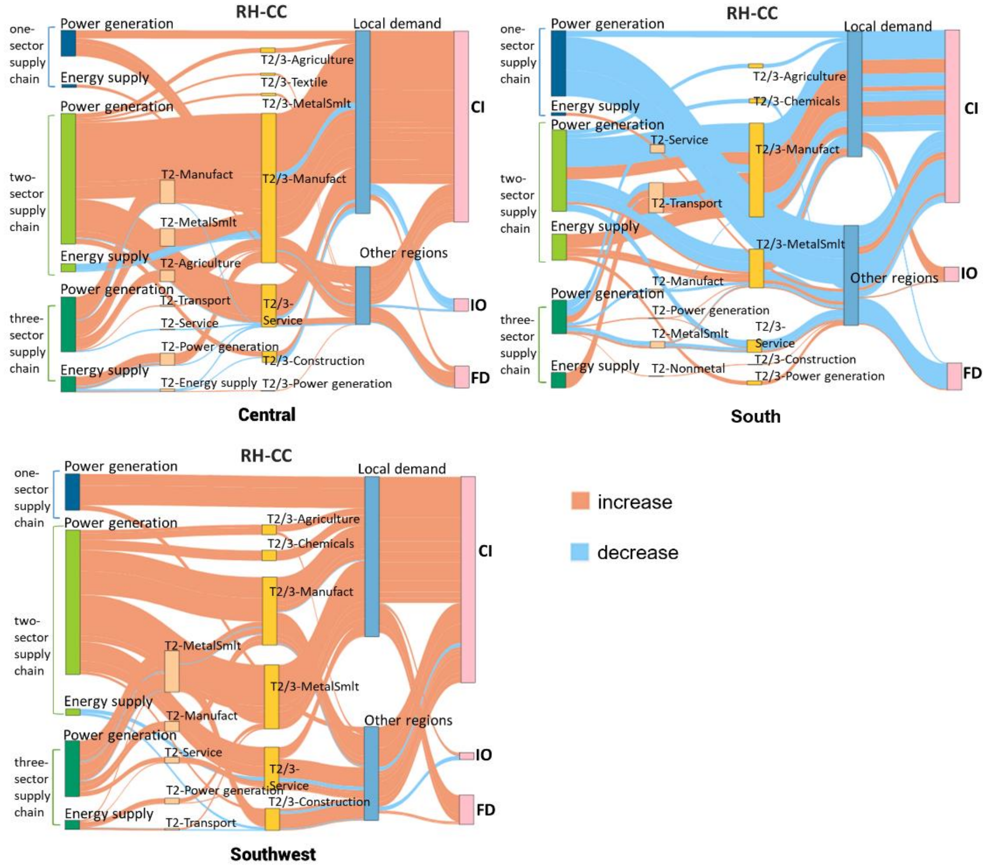

Fig. S9 Illustrative Map of Embodied Coal Consumption Flow Changes between the RH and CC Scenarios for Other Regions in 2060

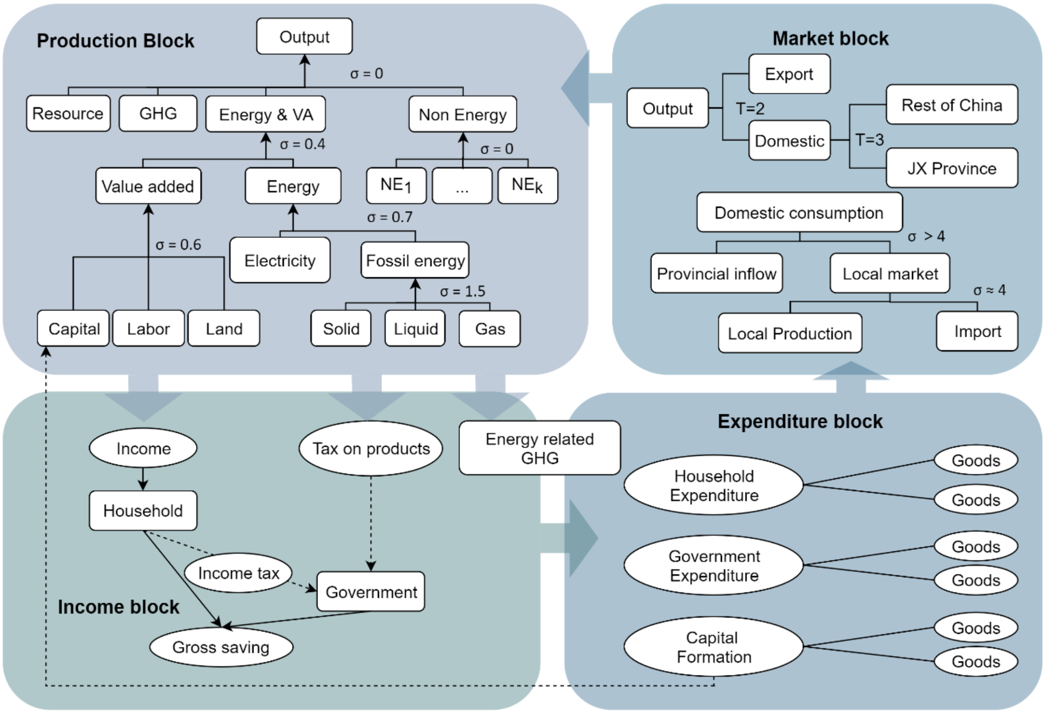

Fig. S10 The four interacting blocks in the IMED|CGE model framework

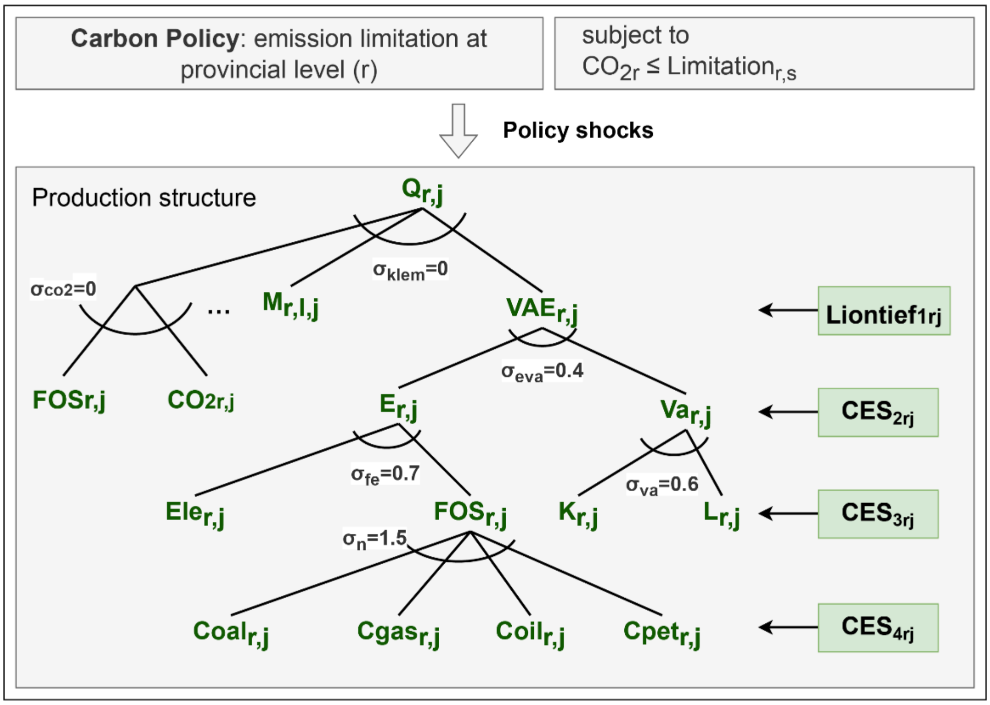

Fig. S11 The nesting structure of production in the IMED|CGE model (r, j, s stand for region, sector, and scenario.)
